# Changes in Body Weight Perception, Lifestyle Habits, and Health Awareness in Croatia: A Comparative Population Survey (2022–2024)

**DOI:** 10.3390/nu18010011

**Published:** 2025-12-19

**Authors:** Sanda Marusic, Radenka Munjas Samarin, Vjekoslav Cigrovski, Silvija Canecki Varzic, Ines Bilic-Curcic, Lana Ruzic, Maja Cigrovski Berkovic

**Affiliations:** 1 Faculty of Kinesiology, University of Zagreb, 10000 Zagreb, Croatia; sanda.marusic1@gmail.com (S.M.); vjekoslav.cigrovski@kif.hr (V.C.); lana.ruzic.svegl@kif.unizg.hr (L.R.); maja.cigrovskiberkovic@gmail.com (M.C.B.); 2 SmartUp, 10000 Zagreb, Croatia; radenka.munjas-samarin@smartup.hr; 3 Department of Endocrinology and Metabolism, University Hospital Center Osijek, 31000 Osijek, Croatia; silvija.canecki@gmail.com; 4 Faculty of Dental Medicine, JJ Strosmayer University, 31000 Osijek, Croatia; 5 Faculty of Medicine, JJ Strosmayer University, 31000 Osijek, Croatia

**Keywords:** Croatia, obesity, overweight, body image, physical activity, diet, public health, prevention, satisfaction

## Abstract

**Background**: Croatia has the highest prevalence of obesity among European Union member states, with more than 65% of adults classified as overweight or obese. Understanding public perceptions and behaviours related to body weight is essential for designing effective prevention and treatment strategies. **Objective**: This study compared Croatian adults’ satisfaction with body weight, physical fitness, and dietary habits in 2022 and 2024, exploring potential behavioural changes following national awareness campaigns and the introduction of new anti-obesity measures. **Methods**: Data were collected via computer-assisted web interviews (CAWI) from representative national samples of adults aged 18 years and older (N = 798; 398 in 2022, 400 in 2024). Analyses used descriptive statistics, Chi-square or Fisher’s exact tests for categorical variables, and independent t-tests for continuous variables. **Results**: In both years, fewer than half of respondents were satisfied with their body weight or physical fitness. Obesity prevalence rose slightly among men (from 18.9% to 25.4%), while rates among women remained stable. Although 93% of women and 78% of men were aware of BMI, only 21% knew their exact value. Individuals with obesity were significantly more dissatisfied with their body shape (*p* < 0.001). One-third reported dieting within the past six months, and only one in ten sought medical advice for weight management. Lack of time and the high cost of healthy foods were the most frequently cited barriers to healthier lifestyles. **Conclusions**: Body weight satisfaction and lifestyle habits among Croatian adults remain suboptimal. Targeted, gender-sensitive, and web-based interventions are needed to promote awareness, improve self-perception accuracy, and enhance obesity prevention efforts.

## 1. Introduction

Obesity affects more than one billion adults worldwide and remains an underdiagnosed health issue. Only about one-third of individuals with obesity receive a formal diagnosis, and even fewer receive appropriate treatment [1]. According to Eurostat, over half of the European Union’s population is overweight, with Croatia recording the highest proportion of adults living with obesity [2]. Obesity develops in two main stages: a preclinical phase, characterised by cellular and tissue changes that lead to structural alterations in organs, and a later stage when organ function becomes impaired. At this point, obesity is considered a chronic disease in its own right and is associated with more than 230 health complications [3]. Individuals with obesity face an increased risk of morbidity and mortality from a wide range of conditions, including diabetes, hypertension, respiratory diseases, and coronary artery disease, all of which substantially increase healthcare costs [4,5]. Moreover, obesity diagnosed in midlife has lasting consequences, including increased frailty in older age [6].

Obesity arises from complex interactions of behavioural, metabolic, and environmental factors. Dietary patterns high in energy density, sugar-sweetened beverages, and processed foods, combined with insufficient physical activity and prolonged sedentary behaviour, are recognised as primary lifestyle drivers of weight gain. Psychosocial factors—including stress, stigma, socioeconomic disadvantage, and cultural norms surrounding body image—also contribute to obesity risk. Large-scale population studies such as the WHO STEPS surveys, the European Health Interview Survey (EHIS), and Eurobarometer reports have extensively explored lifestyle habits, weight perception, and health awareness across Europe [7,8,9].

However, Croatia has been underrepresented in these comparative analyses, and no prior studies have examined changes in weight perception and lifestyle habits over time using nationally representative samples. Croatia’s sociocultural context, characterised by population ageing, regional socioeconomic disparities, and traditionally energy-dense dietary patterns, underscores the importance of monitoring changes in public attitudes and health behaviours.

Excess body weight is typically assessed using body mass index (BMI) and, more accurately, waist circumference [10], which provides a stronger indication of cardiovascular risk. BMI classifications define overweight as 25–30 kg/m^2^ and obesity as above 30 kg/m^2^ [11]. People living with obesity often face stigma, low self-esteem, and denial regarding their condition, all of which can reduce engagement in health-promoting behaviours and worsen health outcomes [12,13]. Labelling individuals as “obese”, even when intended by healthcare professionals to encourage weight loss, has been criticised in many studies for its potential to aggravate psychological distress [14]. As a result, some individuals avoid healthcare appointments, leading to inadequate management of chronic conditions and a greater risk of cardiometabolic complications [15].

Given the magnitude of this issue, obesity prevention and management have become key public health priorities in Croatia. The country has joined broader European and global initiatives aimed at combating the obesity epidemic. In recognition of the growing burden, the Croatian Parliament declared 16 March as the National Day of Obesity Awareness (“Narodne novine,” No. 60/17) to highlight the causes and consequences of obesity and to emphasise prevention, early diagnosis, and treatment [16].

In 2019, data indicated that 65% of Croatian adults had excess body weight, with 23% classified as obese. Projections estimate that by 2030, obesity prevalence could reach 32.4% among men and 30.5% among women [17]. In response, the Croatian Parliament adopted a Resolution on Obesity in April 2023, urging coordinated action from the government, health authorities, educational institutions, and media to strengthen prevention, diagnosis, and treatment.

The resolution highlights the creation of supportive environments in schools and workplaces, improved access to multidisciplinary healthcare teams, and equitable availability of professional guidance and resources. It also calls for increased scientific research, integrated monitoring systems for nutritional status, and evidence-based policymaking. These commitments were further reinforced by the Action Plan for Obesity Prevention 2024–2027, published in March 2024, which focuses on promoting healthy lifestyles and improving the recognition and management of obesity across all age groups.

The present study assessed Croatian adults’ satisfaction with their body weight, fitness, and eating habits in 2022 and 2024. We hypothesised that increased awareness, new therapeutic options, and national policies might influence public attitudes and behaviours related to obesity. Although Croatia participates in international surveillance systems (e.g., EHIS, Eurostat) [8,9], these sources primarily report anthropometric status and selected lifestyle indicators but do not assess individuals’ satisfaction with weight, perceived BMI, or health-seeking behaviour. To date, no repeated, nationally representative surveys have evaluated whether Croatian adults’ attitudes and behaviours toward obesity have shifted during a period of increasing policy activity, including the National Day of Obesity Awareness (2017), the 2023 Parliamentary Resolution, and the 2024–2027 Action Plan. This study therefore provides the first comparative, population-level assessment of attitudinal and behavioural indicators relevant to obesity prevention in Croatia.

## 2. Materials and Methods

### 2.1. Study Design and Participants

This study used online data collection via the Computer-Assisted Web Interviewing (CAWI) method to obtain survey data from the general adult population of Croatia (aged 18 years and over). Participants were recruited through the professional online research panel (CINT), a global leader in digital data collection that complies with international research ethics standards [18] and ISO-certified market research procedures [19]. standards. It maintains a database of individuals who have provided prior consent to be invited to participate in survey-based studies. The panel includes members of the general population and is designed to be nationally representative based on region, age, and gender. Quotas for the present study were applied during recruitment to ensure representativeness across these key demographic variables.

All participants were informed about the purpose and procedures of the study before participation and provided electronic informed consent. Participation was voluntary, and respondents could withdraw at any time without consequence. No personally identifiable information was collected, and all responses were recorded anonymously in accordance with data protection regulations and ethical research standards (e.g., GDPR). As this study involved anonymous, minimal-risk survey research among adults with no collection of identifiable or sensitive data, ethics approval was waived in accordance with national and institutional guidelines.

The final sample comprised 798 participants, surveyed at two time points over a two-year period: 398 participants in 2022 and 400 participants in 2024. This sample represents a cross-section of the Croatian adult population, with a confidence interval of ±4.4%. Quotas for age (18–29, 30–39, 40–49, 50–59, and ≥60 years), gender, and region were applied according to the most recent Croatian Census (2021) to ensure representativeness, resulting in nearly equal proportions of women (53% in 2022; 51.7% in 2024) and men (47% in 2022; 48.3% in 2024).

### 2.2. Questionnaire

Data were collected using a structured, self-administered online questionnaire developed specifically for this study (Upitnik za opću populaciju 2024). The instrument was distributed via the professional online research panel CINT, which maintains a nationally representative database of adults residing in Croatia.

The questionnaire was designed to assess participants’ awareness, attitudes, and behaviours related to body weight, nutrition, and lifestyle, as well as to gather anthropometric and sociodemographic information.

The first section measured satisfaction with body weight, physical fitness, dietary habits, and body image using a five-point Likert scale (1 = “not at all satisfied” to 5 = “completely satisfied”). Participants expressing low satisfaction (responses 1 or 2) were asked to select up to three reasons for dissatisfaction from a predefined list (e.g., lack of time for exercise, cost of healthy food, or health limitations).

Subsequent items assessed recent weight management and health-related behaviours, including dieting, exercise frequency, supplement use, and participation in online communities related to body image or weight loss. Respondents were also asked about their perceived weight status, desired weight change, and willingness to use physician-recommended products for weight reduction (rated on a five-point scale).

Self-reported anthropometric data included body weight (kg), height (cm), and, where known, body mass index (kg/m^2^). Respondents were also asked about their familiarity with the BMI concept and whether they had ever calculated it. Additional questions addressed the presence of chronic conditions (e.g., hypertension, dyslipidaemia, diabetes, chronic pain) and engagement in regular physical activities (e.g., gym training, running, cycling, yoga, or home exercise).

The final section captured demographic characteristics including gender, age, region of residence (by county), employment status, marital status, educational attainment, and net monthly household income.

The questionnaire was developed in Croatian and reviewed by a panel of public health and behavioural science experts to ensure content validity and clarity. To improve internal validity, the questionnaire included several internal consistency indicators. Items addressing body-weight perception, BMI awareness, and satisfaction with appearance were intentionally phrased in parallel structures to identify contradictory responses. Additionally, self-reported height and weight values were cross-referenced with perceived weight status to detect implausible BMI classifications

The questionnaire was pretested on a small sample (*n* = 30) of adults to assess comprehension, response time, and internal consistency. Minor adjustments were made to improve wording and flow before final deployment. The final instrument consisted of 25 items and required approximately 10–12 min to complete.

### 2.3. Statistical Analysis

Data were analysed using IBM SPSS Statistics for Windows, Version 24.0 (IBM Corp., Armonk, NY, USA). Descriptive statistics summarised the basic characteristics of the sample—proportions for categorical data and mean ± standard deviation (SD) for continuous variables such as age and BMI. Comparisons of categorical variables between the 2022 and 2024 samples were performed using the Chi-square test or Fisher’s exact test (used as an alternative to the Chi-square test for 2 × 2 contingency tables). For continuous variables, an independent samples t-test was used after confirmation of homogeneity of variances with Levene’s test. To examine whether the associations between BMI category and (a) satisfaction with body weight and (b) self-perceived excess weight were consistent across survey years, we conducted tests of homogeneity of odds ratios using the Breslow–Day test for 2 × k tables. For three-way contingency tables (BMI × response category × year), log-linear modelling was applied to assess the presence of higher-order interactions. Statistical significance was set at *p* < 0.05.

## 3. Results

### 3.1. Demographic Characteristics of Participants

In 2022 and 2024, quotas for the general population are similar, with no statistically significant deviation in sample structure: 53% of respondents were female in 2022 and 52% in 2024, while 51% and 52% were aged between 30 and 49 years. Detailed characteristics of participants are presented in Table 1.

### 3.2. Body Weight Distribution and BMI Trends

**Figure 1:**
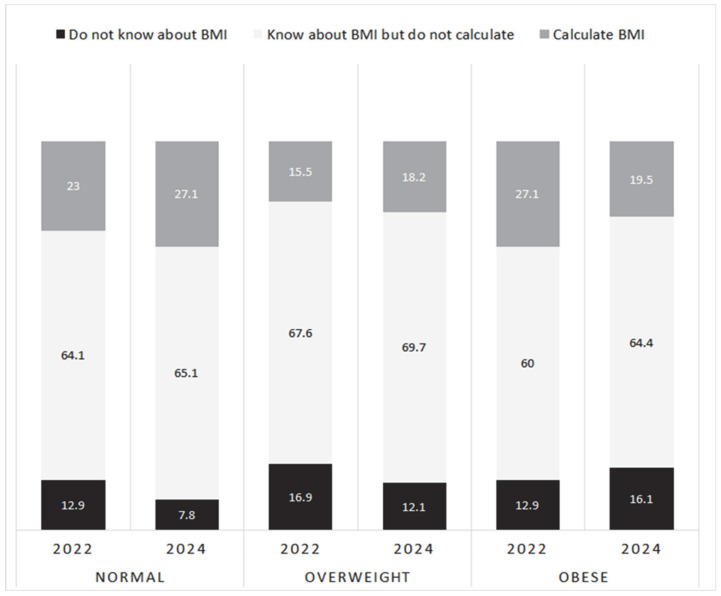
Proportion of participants aware of BMI according to weight category, 2022 and 2024.

In 2022, 93% of females and 78% of males were familiar with the concept of body mass index (BMI); however, most did not calculate it (65% overall). Only 21% could report their BMI, with a mean value of 25.86 ± 5.23 kg/m^2^ (27.34 ± 5.27 kg/m^2^ for males; 24.97 ± 5.60 kg/m^2^ for females). Participants aware of and calculating their BMI were most often of normal weight, whereas those unaware were predominantly classified as obese (*p* < 0.05; Figure 1). In 2024, the proportion of respondents with obesity who were unfamiliar with BMI rose from 12.9% to 16.1%, while the proportion who were aware and had calculated their BMI decreased from 27.1% to 19.5% (*p* = 0.441).

Among men, BMI categories in 2022 were as follows: underweight (2.2%), normal weight (34.1%), overweight (44.9%), and obesity (18.9%). In 2024, these shifted slightly to 2.1%, 34.2%, 38.3%, and 25.4%, respectively. Among women, the proportions in 2022 were 4.3%, 51.0%, 28.1%, and 16.7% compared with 4.9%, 48.5%, 28.2%, and 18.4% in 2024. The mean BMI increased modestly among men (26.6 ± 4.64 to 27.16 ± 5.10 kg/m^2^) and slightly among women (25.2 ± 4.97 to 25.68 ± 5.94 kg/m^2^). Although the gender difference in mean BMI was statistically significant in 2022 (*p* = 0.004), it was not in 2024 (*p* = 0.052). Overall, obesity prevalence and mean BMI rose slightly among men, while values among women remained stable.

### 3.3. Satisfaction with Body Appearance

Participants’ satisfaction with body appearance was assessed in both 2022 and 2024 (Table 2). In 2022, 47.2% of respondents reported feeling satisfied or neutral about their appearance, while 16.3% were undecided (*p* < 0.05). In contrast, 39.2% perceived themselves as having excess weight, with no significant gender differences (*p* > 0.05). This trend persisted in 2024.

Nearly all respondents with obesity were aware of their condition. However, only about half (50%) of those classified as overweight correctly recognised their weight status, while 44% believed they were not overweight (*p* < 0.05, excluding “don’t know” responses). Similar patterns were observed in 2024, with more detailed results by gender presented in Table 2.

The Breslow–Day test showed that the association between gender and satisfaction with body weight did not differ significantly between 2022 and 2024 (*p* > 0.05), indicating stable gender-related patterns across years. The same was observed for the association between gender and perceived excess weight (*p* > 0.05).

### 3.4. Satisfaction with Body Appearance According to Weight Category

Satisfaction with body weight differed significantly across BMI categories in both survey years (Table 3a,b). In 2022, dissatisfaction increased progressively from normal weight (21.8%) to overweight (33.8%) and obesity (77.1%; *p* < 0.001). A comparable pattern was observed in 2024 (16.3%, 45.5%, and 81.6%, respectively; *p* < 0.001).

Perceived excess weight also showed a strong association with BMI. In 2022, 98.6% of respondents with obesity and 50.0% of those with overweight indicated they had excess weight, compared with 8.8% of normal-weight individuals. Similar distributions were found in 2024 (94.3%, 55.3%, and 9.0%).

To assess whether these BMI-related associations changed between survey years, log-linear analysis was used to test for higher-order interactions (BMI × response × year). For satisfaction with body weight, combinations of normal BMI with “satisfied” responses were significantly overrepresented in both 2022 (OR = 13.35, *p* < 0.001) and 2024 (OR = 14.66, *p* < 0.001). No significant three-way interaction was detected, indicating a stable association across years.

For perceived excess weight, “no excess weight” among normal-weight respondents showed consistently high likelihood (2022: OR = 27.9; 2024: OR = 26.7; both *p* < 0.001), while “yes” responses were overrepresented among overweight and obese groups. Again, no significant three-way interaction emerged, indicating that the relationship between BMI and perceived excess weight remained unchanged between the two survey waves.

### 3.5. Weight-Management Behaviours

Approximately one-third of the population reported following some form of a diet in the previous six months in both 2022 and 2024 (Table 4a,b). A significantly higher proportion of respondents with obesity (42.9%) engaged in dieting compared to those with normal BMI (26.5%, *p* < 0.05). About 20% of participants attempted to increase muscle mass through dietary changes.

Two-thirds of respondents in both years failed to meet the World Health Organization’s (WHO) recommended levels of physical activity. The use of over-the-counter (OTC) weight reduction products and homemade preparations remained stable between 2022 and 2024. In 2022, a greater proportion of individuals with overweight (27.5%) reported using homemade products than those with normal weight (10.6%, *p* < 0.05), but this difference was not present in 2024 (*p* = 0.324).

Regarding the sources of information, 35.7% of respondents in 2022 sought advice on healthy BMI from online forums or social media groups—significantly more women (43.6%) than men (26.7%, *p* < 0.05). By 2024, this number declined (*p* = 0.053). In contrast, only 9.3% of participants in 2022 sought expert medical advice about body weight (12.8% of men and 6.2% of women, *p* < 0.05). By 2024, consultation rates with healthcare professionals increased modestly, especially among individuals with obesity (*p* < 0.001).

### 3.6. Physical Fitness and Eating Habits

**Figure 2:**
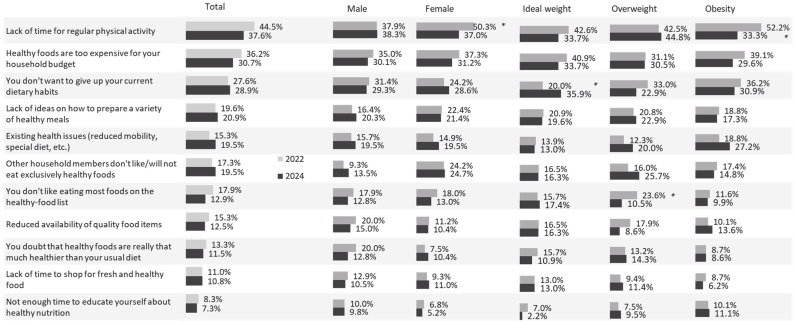
Reasons for dissatisfaction on total sample, as well as according to gender and BMI, in 2022 and 2024. * *p* < 0.05.

About 40% of the adult population expressed dissatisfaction with their physical fitness, and dissatisfaction increased with BMI (31% among normal-weight individuals, 36% overweight, 69% obese; *p* < 0.05). Interestingly, nearly 30% of participants with obesity reported being satisfied with their fitness level (Figure 2).

Roughly one-third (32%) of respondents engaged in regular physical exercise—defined as at least 30 min per session, five times per week—with slightly more men than women participating (*p* > 0.05). Although physical activity tended to decline with increasing BMI, the difference was not statistically significant (*p* > 0.05).

Regarding dietary habits, 41% of respondents were satisfied with their eating patterns. Individuals with obesity were significantly more dissatisfied than those with normal or overweight BMI (*p* < 0.05). However, 24% of participants with obesity reported no dissatisfaction with their die (Figure 2).

The main source of dissatisfaction with physical fitness was lack of time for regular activity (45% overall; 50% of women vs. 38% of men, *p* < 0.05). Dissatisfaction with eating habits was most commonly attributed to the financial inaccessibility of healthy foods (36%), followed by unwillingness to change existing habits (28%). Other barriers included lack of ideas for preparing healthy meals (20%) and family members’ aversion to healthy foods (17–18%).

Men were significantly more likely than women to doubt whether “healthy” foods were truly beneficial (20% vs. 7%, *p* < 0.05) and to perceive them as less available (20% vs. 11%, *p* < 0.05). Respondents with excess weight reported more difficulty changing eating habits than those of normal weight (33–36% vs. 20%, *p* < 0.05).

### 3.7. Health Conditions

**Figure 3:**
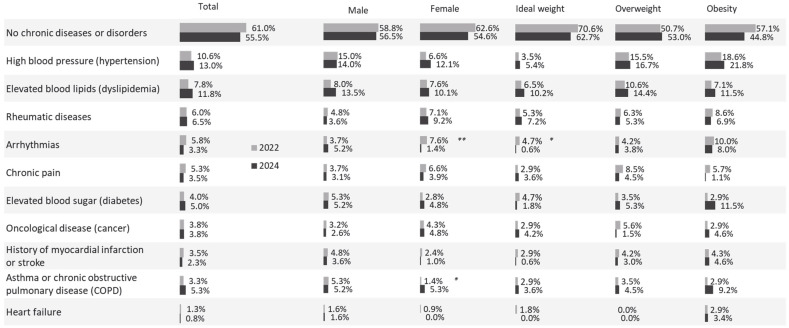
Health conditions on total sample, as well as according to gender and BMI, in 2022 and 2024. * *p* < 0.05. ** *p* < 0.01.

Overall, 61% of participants reported no chronic conditions. The most common disorders were hypertension (11%), hyperlipidaemia (8%), locomotor pain or chronic musculoskeletal disorders (6%), and diabetes mellitus (4%). Participants with normal BMI had significantly fewer chronic conditions than those with obesity (*p* < 0.05). Hypertension, hyperlipidaemia, chronic pain, and diabetes were all more prevalent among individuals with higher BMI. Additionally, arterial hypertension was more common among men (15%) than women (7%, *p* < 0.05) (Figure 3).

### 3.8. Differences Between 2022 and 2024

Most measured behaviours and perceptions remained stable between the two survey years. No significant differences were found in dieting frequency, use of OTC products, use of homemade preparations, muscle-building dietary efforts, or engagement in regular physical activity (all *p* > 0.05) (Table 5). The only statistically significant change was a reduction in the proportion of respondents seeking weight-management information on online forums or social media groups, which decreased from 35.7% in 2022 to 30.0% in 2024 (*p* < 0.05). Consultations with healthcare professionals increased modestly (from 9.3% to 11.5%), though not significantly (*p* = 0.196).

## 4. Discussion

Despite scientific debates on nomenclature, excess weight (overweight and obesity) is an underestimated global problem that adversely affects various health outcomes [20]. According to the World Health Organization (WHO), the risk of obesity-related comorbidities increases with BMI and is already present in individuals with overweight (BMI between 25 and 30 kg/m^2^) [17]. Moreover, negative perception of one’s body image related to obesity interferes with quality of life [21]. A key contribution of our study lies in providing the first longitudinal comparison of obesity-related perceptions and behaviours in Croatia across a period of national policy activity. Existing Croatian and EU monitoring datasets do not capture psychosocial indicators such as body satisfaction, perceived weight status, willingness to seek professional advice, or perceived barriers to healthy lifestyles. Our findings therefore fill an important knowledge gap by documenting stable patterns that national statistics alone cannot reveal.

Our results show that 36% of the Croatian general population is satisfied with their body mass, while, according to Eurostat data from 2023, 58% of females and 73% of adult males in Croatia are classified as overweight [2]. Specifically, 67% of participants in the overweight category (average BMI of 27 kg/m^2^) and 23% of participants in the obese category (average BMI of 33 kg/m^2^) reported satisfaction with their body mass. Positive body image is an important issue to consider when addressing obesity. It is imperative to develop and maintain, as negative body image has far-reaching consequences that affect mental health and can lead to eating disorders, further complicating excess weight management [21].

Indeed, 58% of our respondents, regardless of their BMI, do not consider their body weight excessive. This discrepancy between actual and perceived body weight is particularly notable among participants classified as overweight, where 50% are unaware of their excess body weight, even though their calculated BMI places them in this category. The finding that approximately half of individuals classified as overweight do not recognise their excess weight suggests a sociocultural normalisation of heavier body sizes in Croatia, consistent with trends in regions with high obesity prevalence. Psychological mechanisms such as stigma avoidance and optimistic bias may also promote denial or minimization of excess weight. This misperception has direct public-health consequences: individuals unaware of their excess weight are less likely to initiate lifestyle changes or seek medical advice.

As the prevalence of obesity continues to rise, individuals with overweight are likely to have obesity in the future. According to published literature, misperception of excess body weight is reported in up to 22% of adults, which, based on our results, may be an underestimated issue. Weight misreporting may contribute to the pronounced gap between measured BMI categories and perceived weight, particularly among individuals with overweight. Overreporting of physical activity may also contribute to the apparent inconsistency between high rates of inactivity and the relatively high proportion reporting satisfaction with fitness. Underrecognition or underreporting of chronic diseases, especially among individuals not engaged with healthcare providers, may underestimate the true burden of obesity-related comorbidities.

This is an important consideration when planning health-related interventions, as perceptions of body image can influence modifiable health risks, such as dietary choices and physical activity [22]. Public health campaigns should focus on promoting a positive energy balance by encouraging healthier eating habits, reducing excess energy intake, and increasing energy expenditure [23]. From this perspective, providing opportunities for recreational participation in various sports from an early age, rather than focusing solely on competitive sports, may help develop healthy habits and support the achievement and maintenance of a healthy weight [24].

Furthermore, special attention should be given to people with subclinical excess weight, as effective health strategies could prevent functional organ damage [25]. Given that only 9–11% of respondents consulted healthcare professionals, misperception may further delay diagnosis and management of cardiometabolic risk, which is the leading cause of obesity-related morbidity and mortality. Moreover, this leads to another important issue the health care system must address—how and where people seek help for achieving a healthy weight. Our participants wished to lose an average of 11 kg, but this desire increased to 22 kg among individuals living with obesity. Regarding health information, 36% sought advice from forums (44% of women vs. 27% of men), while only 9% consulted medical professionals (13% of men vs. 6% of women, *p* < 0.05). De Heer and colleagues reported similar findings based on an analysis of cross-sectional data from the National Health and Nutrition Examination Survey (NHANES), where only 10.9% of adults with excess weight sought help with weight management from a health professional, even though weight loss advice from a health care provider is associated with greater odds of achieving it [26]. The literature also suggests that among adults living with obesity, women aged 40 to 49 with chronic conditions are more likely to receive advice to lose weight from healthcare professionals, mostly related to lifestyle measures, particularly when the need to lose up to 21% of body weight is identified [27]. Our results, like others, suggest men are less likely to perceive weight problems [28]. Interestingly, when seeking advice, men are more likely to contact healthcare professionals and less likely to use over-the-counter or homemade products or forums, regardless of their health status.

Therefore, it seems prudent to develop different strategic approaches for women and men living with obesity, especially as evidence shows that women are at higher risk of developing obesity-related physical and psychological comorbidities and have twice the mortality risk [29,30]. As our data suggest, regardless of health status, women are more likely to seek non-professional advice and participate in various forums or groups focused on physical appearance and weight loss. This is potentially dangerous but also presents an opportunity for a personalised approach. These findings support the need for gender-specific interventions—for example, clinician-led online platforms for women and structured physical activity programmes tailored for men. Gender differences observed in this study align with broader sociocultural patterns. Women’s higher use of online forums may reflect greater societal pressure related to appearance, higher engagement in peer-driven support systems, and a tendency to seek community-based guidance rather than professional advice. In contrast, men’s greater likelihood of consulting healthcare professionals may stem from gendered norms that frame weight concerns as health-related rather than appearance-related. Gender-specific misperception of weight—whereby men underrecognize excess weight more frequently—may delay men’s engagement in preventive behaviours, underscoring the need for gender-tailored interventions.

Physical activity patterns showed that only a third of the population engaged in regular exercise, defined as 30 min sessions five times a week. Unfortunately, failure to meet physical activity recommendations and a sedentary lifestyle are closely linked to excess weight and chronic non-communicable diseases [31]. Cardiorespiratory fitness is a measure of both and is directly negatively correlated with morbidity and mortality [32]. A significant 40% of participants were dissatisfied with their physical fitness, which correlated with higher BMI levels (31% of respondents with normal BMI, 36% with overweight, and 69% living with obesity, *p* < 0.05). Therefore, addressing physical fitness as a weight loss intervention could be an important tool. Published research suggests that interventions targeting physical activity are likely to be more effective for men than for women as part of weight loss programmes. Although men are harder to recruit for such programmes, once included, they are less likely to drop out, which is also an interesting finding from the perspective of healthy weight maintenance [28].

The primary barrier to improved physical fitness was lack of time, particularly by women. Therefore, technology-assisted programmes (web-based) or intensity-alternating exercises such as HIIT (high-intensity interval training), which are suitable for shorter durations and for people without comorbidities, could be a potential solution [33]. This is also supported by a Cochrane meta-analysis, which suggests that interactive computer-based interventions, compared to no or minimal intervention (such as pamphlets or usual care), are effective and feasible for enhancing weight loss and maintenance [34,35].

Regarding dietary habits, 41% of participants reported satisfaction; however, dissatisfaction was higher among individuals living with obesity than among those with overweight or of normal weight (*p* < 0.05). The main barriers to healthy eating were financial constraints, lack of meal preparation ideas, and a preference for unhealthy foods by both participants and their families. Robertson et al. similarly found that the social role of food is important for maintaining relationships, while also serving as a significant barrier to weight loss [28]. Thus, family-based dietary interventions, which have already demonstrated long-term effectiveness for children living with obesity, could be a valuable option for adults living with partners, spouses, or families [36]. Furthermore, Winston and colleagues highlight the importance of supportive peers in achieving more successful weight loss among adults with obesity [37]. Data from an umbrella review of family-based interventions targeting weight management in children with weight problems also suggest these interventions are effective in improving child weight [38]. Comparable results have been reported in research on successful behavioural changes when partners are involved [39]. Socioeconomic disparities emerged indirectly in this study. Participants reporting dissatisfaction with their diet most frequently cited financial inaccessibility of healthy foods, consistent with research showing that lower-income households face disproportionate barriers to adopting healthier eating patterns. Socioeconomic status (SES) may also influence weight perception, as financial constraints can affect norms around body weight and limit investments in fitness or structured exercise. Similarly, the cost of facilities or equipment may restrict opportunities for physical activity. Future analyses incorporating SES stratification are warranted to better understand these structural determinants.

Healthwise, 61% of interviewed participants reported no chronic conditions. However, those with a normal BMI had significantly fewer chronic diseases than participants with obesity. Conditions such as elevated blood pressure, hyperlipidaemia, chronic pain, and diabetes were more prevalent among individuals with higher BMI (*p* < 0.05) [40,41].

Despite the implementation of the National Day of Obesity Awareness, the 2023 Parliamentary Resolution on Obesity, and the 2024–2027 Action Plan, no substantial improvements were observed in body weight satisfaction, physical activity, or healthier eating patterns between 2022 and 2024. Awareness of BMI remained high, yet this did not lead to increased self-monitoring or professional help-seeking, as only one in ten participants consulted healthcare providers. This suggests that while national strategies have increased visibility and public discourse around obesity, their impact on individual behavioural change remains limited. The persistence of financial barriers to healthy eating and lack of time for physical activity further indicates that national strategies must go beyond awareness-raising to create supportive environments and structural changes that enable healthier choices.

Although the study coincides with major national initiatives, the absence of significant differences between 2022 and 2024 should not be interpreted as evidence that policies were ineffective. Given the short time between the adoption of the 2023 Resolution, release of the 2024–2027 Action Plan, and the second survey wave, population-level behavioural change may not have yet occurred. Additionally, awareness campaigns generally influence knowledge before behaviour, and structural factors such as food prices and time constraints—both of which remained substantial barriers—may limit behavioural translation. Thus, the observed stability reflects the early stage of national policy implementation rather than its eventual long-term impact.

### Limitations and Strengths of the Study

This study offers valuable insights into body weight satisfaction, physical fitness, and dietary habits among adults in Croatia, a country with one of the highest rates of excess body weight in the European Union. However, several limitations should be acknowledged.

First, the study used BMI as the primary measure of adiposity. Although BMI is a practical and widely used index, it does not reflect body composition or fat distribution, both of which are important for assessing metabolic health. Second, the measures of physical fitness and dietary habits were self-reported, which may introduce recall bias and social desirability bias, as participants might underreport or overreport certain behaviours. Third, although the sample included individuals with chronic diseases, the study did not distinguish between specific conditions or assess their severity or impact on satisfaction levels. Furthermore, the two survey waves (2022 and 2024) provide temporal comparison but are not designed as formal policy-evaluation studies, and due to descriptive and cross-sectional design, causal inference regarding the effects of national policy measures cannot be drawn.

In addition, the reliance on self-reported anthropometrics may have introduced systematic bias, as individuals tend to underreport weight and overreport height, potentially leading to BMI misclassification. Similarly, physical activity is frequently overestimated in self-report formats, which may partially explain discrepancies between reported activity levels and dissatisfaction with fitness. Chronic conditions may also be underreported, especially among individuals without regular medical follow-up. These biases may result in conservative estimates of obesity prevalence and may influence observed associations with satisfaction or health awareness.

Despite these limitations, the study has notable strengths. It used a representative sample of the Croatian adult population, stratified by age, gender, and region, enhancing generalisability. The use of a standardised and GDPR-compliant online methodology ensured anonymity and minimised interviewer bias. Importantly, the study addresses a critical knowledge gap by providing comparative data over time (2022 vs. 2024), enabling the observation of trends and potential behavioural shifts in response to national policies and public awareness campaigns.

The practical implications of these findings are multifactorial. Given the stable prevalence of excess weight and the persistent gap between objective and perceived body weight, public health strategies should prioritise improving health literacy and promoting accurate self-assessment of weight status. Gender differences observed in help-seeking behaviour suggest the need for gender-tailored interventions—for example, clinician-guided digital programmes for women, and structured physical activity–based initiatives for men. Financial barriers to healthy eating indicate that policy-level measures, such as subsidies for nutrient-dense foods or municipal programmes supporting community-based exercise opportunities, may be required. Integrating systematic weight-management counselling into primary care, supported by digital self-monitoring tools, could enhance early intervention. These approaches align with Croatia’s 2024–2027 Action Plan for Obesity Prevention and may support more effective translation of national policies into behavioural change.

## 5. Conclusions

Despite comprehensive national initiatives, satisfaction with body weight, physical fitness, and dietary habits among Croatian adults remains low. Obesity prevalence continues to rise among men, and professional engagement in weight management is rare.

National policies have improved awareness but not behaviour. To achieve population-level impact, future interventions must integrate multidisciplinary counselling into primary care, reduce economic barriers to healthy eating, and develop personalised, digital, and gender-sensitive prevention programmes.

Longitudinal monitoring is essential to evaluate the long-term effects of Croatia’s Action Plan for Obesity Prevention 2024–2027 and to guide future evidence-based policy adjustments.

## Figures and Tables

**Table 1:** Participants’ characteristics in 2022 and 2024.

		Year			
		2022		2024	
		*n*	(%)	*n*	(%)
Gender	Male	187	(47.0)	193	(48.3)
	Female	211	(53.0)	207	(51.7)
Age	18–29	90	(22.6)	79	(19.8)
	30–39	109	(27.4)	112	(28.0)
	40–49	93	(23.4)	98	(24.5)
	50–59	72	(18.1)	76	(19.0)
	60+	34	(8.5)	35	(8.8)
BMI	underweight	13	(3.3)	14	(3.5)
	normal weight	170	(42.8)	166	(41.6)
	overweight	142	(35.8)	132	(33.1)
	obesity	72	(18.1)	87	(21.8)
Region	Zagreb and surroundings	105	(26.4)	115	(28.7)
	Northern Croatia	70	(17.6)	70	(17.5)
	Slavonia	69	(17.3)	67	(16.8)
	Lika, Kordun, Banovina	33	(8.3)	30	(7.5)
	Istria, Primorje, Gorski Kotar Kotar	49	(12.3)	51	(12.8)
	Dalmatia	72	(18.1)	67	(16.8)
Employment	employed full time	260	(66.7)	259	(64.8)
	employed part-time	19	(4.9)	19	(4.8)
	farmer	1	(0.3)	1	(0.3)
	unemployed	28	(7.2)	45	(11.3)
	student	28	(7.2)	21	(5.3)
	retired	43	(11.0)	43	(10.8)
	housewife	11	(2.8)	8	(2.0)
	other			4	(1.0)
Marital status	single	97	(24.4)	111	(27.8)
	married/lives with a partner	267	(67.1)	244	(61.0)
	divorced	24	(6.0)	23	(5.8)
	widow/er	4	(1.0)	15	(3.8)
	NA	6	(1.5)	7	(1.8)
Education	elementary school	4	(1.1)	7	(1.8)
	3-year high-school	43	(10.8)	39	(9.8)
	4-year high school	149	(37.4)	154	(38.5)
	higher education (college)	136	(34.2)	168	(42.0)
	master of science or PhD	63	(15.8)	31	(7.8)
	NA	3	(0.8)	1	(0.3)
Nett household income	up to 333 Eur	22	(5.5)	31	(7.8)
	334–533 Eur	14	(3.5)	28	(7.0)
	534–800 Eur	46	(11.6)	60	(15.0)
	801–1067 Eur	46	(11.6)	48	(12.0)
	1068–1333 Eur	55	(13.8)	70	(17.5)
	1334–1600 Eur	63	(15.8)	71	(17.8)
	1601–2133 Eur	82	(20.6)	61	(15.3)
	2134–2667 Eur	39	(9.8)	31	(7.8)
	more than 2667 Eur	31	(7.8)		

**Table 2:** Participants’ satisfaction with body appearance according to gender.

		2022			2024		
		Male (N = 187) (%)	Female (N = 211) (%)	*p*	Male (N = 193) (%)	Female (N = 207) (%)	*p*
Satisfaction with body weight	Not satisfied	29.9	42.2	0.004 *	33.2	47.3	<0.001
	Undecided	21.9	11.4		16.1	9.7	
	Satisfied	48.1	46.4		50.8	43.0	
Believe to have excess weight	Yes	35.8	42.2	0.432	45.6	40.6	0.206
	No	61.5	55.5		53.4	56.0	
	Don’t know	2.7	2.4		1.0	3.4	

* *p* < 0.05.

**Table 3:** (**a**) Participants’ satisfaction with body appearance according to weight category. (**b**) Participants’ satisfaction with body appearance: 2022 vs. 2024 comparison.

(**a**)									
		**2022**				**2024**			
		**Normal Weight** **N = 170** **%**	**Over-Weight** **N = 142** **%**	**Obesity** **N = 70** **%**	***p***	**Normal Weight** **N = 166** **%**	**Over-Weight** **N = 132** **%**	**Obesity** **N = 87** **%**	***p***
Satisfaction with body weight	Not satisfied	21.8	33.8	77.1	<0.001	16.3	45.5	81.6	<0.001
	Undecided	11.8	22.5	17.1		9.0	19.7	9.2	
	Satisfied	66.5	43.7	5.7		74.7	34.8	9.2	
Believe to have excess weight	Yes	8.8	50.0	98.6	N.A. *	9.0	55.3	94.3	N.A. *
	No	90.0	44.4	1.4		88.0	41.7	5.7	
	Don’t know	1.2	5.6	0		3.0	3.0	0	
(**b**)									
				**2022** **N = 398 (%)**		**2024** **N = 400 (%)**			***p***
Believe to have excess weight		Yes		39.2		43.0			0.548
		No		58.3		54.8			
		Don’t know		2.5		2.3			
Satisfaction with body weight		Not satisfied		36.4		40.5			0.269
		Undecided		16.3		12.8			
		Satisfied		47.2		46.8			

* N.A.: Not available for calculation due to empty cells (frequencies < 1).

**Table 4:** (**a**) Participants using different means (diets, drugs, homemade preparations, advice) to lose weight in the last 6 months according to gender in 2022 and 2024. (**b**) Participants using different means (diets, drugs, homemade preparations, advice) to lose weight in the last 6 months, according to their weight in 2022 and 2024.

(**a**)									
				**2022**			**2024**		
				**M** **N = 187** **(%)**	**F** **N = 211** **(%)**	***p***	**M** **N = 193** **(%)**	**F** **N = 207** **(%)**	***p***
Tried some form of restrictive diet		Yes		27.3	38.4	0.063	31.1	35.7	0.615
		No		67.9	57.3		66.8	62.3	
		Don’t know		4.8	4.3		2.1	1.9	
Tried to increase muscle mass with diet		Yes		25.1	15.6	0.061	17.1	14.5	0.712
		No		71.7	81.0		80.3	82.1	
		Don’t know		3.2	3.3		2.6	3.4	
Exercised at least 5 x/w for 30 min or longer		Yes		36.9	27.5	0.127	28.0	22.7	0.408
		No		59.9	68.2		65.8	72.0	
		Don’t know		3.2	4.3		6.2	5.3	
Used OTC drugs for body weight reduction		Yes		10.7	6.6	0.188	7.3	9.2	0.433
		No		84.5	90.5		90.7	87.0	
		Don’t know		4.8	2.8		2.1	3.9	
Used homemade preparations reduction		Yes		17.1	19.0	0.475	17.1	23.2	0.280
		No		75.9	76.8		78.2	73.4	
		Don’t know		7.0	4.3		4.7	3.4	
Tried to get information on forums or in groups		Yes		26.7	43.6	<0.05	24.4	35.3	<0.05
		No		66.3	52.1		72.5	61.4	
		Don’t know		7.0	4.3		3.1	3.4	
Tried to get expert medical advice		Yes		12.8	6.2	<0.05	14.0	9.2	0.178
		No		84.0	88.2		84.5	87.4	
		Don’t know		3.2	5.7		1.6	3.4	
(**b**)									
		**2022**				**2024**			
		**Normal** **N = 170** **%**	**Over-Weight** **N = 142** **%**	**Obese** **N = 70** **%**	***p***	**Normal** **N = 162** **%**	**Over-Weight** **N = 132** **%**	**Obese** **N = 87** **%**	***p***
Tried some form of restrictive diet	Yes	26.5	38.0	42.9	0.049	28.3	32.6	48.3	<0.05
	No	68.8	56.3	55.7		71.1	65.9	47.1	
	Don’t know	4.7	5.6	1.4		0.6	1.5	4.6	
Tried to increase muscle mass with diet	Yes	22.9	17.6	17.1	0.760	21.1	10.6	12.6	0.083
	No	74.1	78.9	80.0		76.5	85.6	86.2	
	Don’t know	2.9	3.5	2.9		2.4	3.8	1.1	
Exercised at least 5 x/w for 30 min or longer	Yes	34.7	32.4	25.7	0.679	30.1	19.7	21.8	0.192
	No	61.2	64.8	70.0		63.9	73.5	74.7	
	Don’t know	4.1	2.8	4.3		6.0	6.8	3.4	
Used OTC drugs for body weight reduction	Yes	8.8	9.2	8.6	0.973	5.4	6.1	16.1	<0.05
	No	88.2	86.6	88.6		91.0	90.9	82.8	
	Don’t know	2.9	4.2	2.9		3.6	3.0	1.1	
Used homemade preparations reduction	Yes	10.6	27.5	17.1	0.003	16.9	19.7	25.3	0.324
	No	82.4	67.6	80.0		80.7	75.0	70.1	
	Don’t know	7.1	4.9	2.9		2.4	5.3	4.6	
Tried to get information on forums or in groups	Yes	26.7	36.6	44.3	0.309	30.1	28.0	34.5	0.277
	No	66.3	58.5	48.6		68.7	66.7	62.1	
	Don’t know	7.0	4.9	7.1		1.2	5.3	3.4	
Tried to get expert medical advice	Yes	12.8	8.5	15.7	0.336	9.0	6.1	23.0	<0.05
	No	84.0	87.3	81.4		89.8	90.2	75.9	
	Don’t know	3.2	4.2	2.9		1.2	3.8	1.1	

**Table 5:** Differences between 2022 and 2024 in the use of various means (diets, drugs, homemade preparations, advice) to lose weight in the last six months.

		2022 N = 398 (%)	2024 N = 400 (%)	*p*
Tried some form of restrictive diet	Yes	33.2	33.5	0.132
	No	62.3	64.5	
	Don’t know	4.5	2.0	
Tried to increase their muscle mass through diet	Yes	20.1	15.8	0.260
	No	76.6	81.3	
	Don’t know	3.3	3.0	
Exercised at least 5 times a week for half an hour or longer	Yes	31.9	25.3	0.067
	No	64.3	69.0	
	Don’t know	3.8	5.8	
Used over-the-counter (OTC) drugs for body weight reduction	Yes	8.5	8.3	0.821
	No	87.7	88.8	
	Don’t know	3.8	3.0	
Used homemade preparations for body weight reduction	Yes	18.1	20.3	0.479
	No	76.4	75.8	
	Don’t know	5.5	4.0	
Tried to get information on forums or in groups	Yes	35.7	30.0	<0.05
	No	58.8	66.8	
	Don’t know	5.5	3.3	
Tried to get expert medical advice	Yes	9.3	11.5	0.196
	No	86.2	86.0	
	Don’t know	4.5	2.5	

## Data Availability

The original contributions presented in this study are included in the article. Further inquiries can be directed to the corresponding author.
